# Can unhealthy food purchases at checkout counters be discouraged by introducing healthier snacks? A real-life experiment in supermarkets in deprived urban areas in the Netherlands

**DOI:** 10.1186/s12889-020-08608-6

**Published:** 2020-04-21

**Authors:** Marlijn Huitink, Maartje P. Poelman, Jacob C. Seidell, Milan Pleus, Tom Hofkamp, Carlijn Kuin, S. Coosje Dijkstra

**Affiliations:** 1 Department of Health Sciences, Faculty of Science, Vrije Universiteit Amsterdam, Amsterdam Public Health research institute, De Boelelaan 1085, 1081 HV Amsterdam, the Netherlands; 2 Chairgroup Consumpion and Healthy Lifestyles, Wageningen University & Research, Hollandseweg 1, 6706 KN Wageningen, the Netherlands; 3 Nederlandse Zorgautoriteit, Newtonlaan 1, 3584 BX Utrecht, The Netherlands; 4 Sustainability Department, Ahold Delhaize, Provincialeweg 11, 1506 MA Zaandam, The Netherlands; 5 Department of Healthy living, Diabetes Fonds, Amersfoort, The Netherlands

**Keywords:** Supermarkets, Checkout counter, Purchase behavior, Food purchases, Snacks, Impulsive behavior, Food environment

## Abstract

**Background:**

The checkout area in supermarkets is an unavoidable point of purchase where impulsive food purchases are likely to be made. However, the product assortment at the checkout counters is predominantly unhealthy. The aim of this real life experiment was to investigate if unhealthy food purchases at checkout counters in supermarkets in deprived urban areas in the Netherlands can be discouraged by the introduction of the Healthy Checkout Counter (HCC). In addition, we examined customers’ perceptions towards the HCC.

**Methods:**

The HCC was an initiative of a leading supermarket chain in the Netherlands that consisted of displays with a selection of healthier snacks that were placed at the checkouts. We used a real life quasi-experimental design with 15 intervention and 9 control supermarkets. We also performed a cross-sectional customer evaluation in 3 intervention  supermarkets using oral surveys to investigate customers' perceptions towards the HCC (n=134). The purchases of unhealthy and healthier snacks at checkouts were measured with sales data.

**Results:**

During the intervention period, customers purchased on average 1.7 (SD: 0.08) unhealthy snacks per 100 customers in the intervention supermarket and 1.4 (SD: 0.10) in the control supermarket. Linear regression analyses revealed no statistically significant difference in the change during the control and intervention period of sales of unhealthy snacks between the control and intervention supermarkets (B = − 0.008, 95% CI = − 0.15 to 0.14). The average number of healthier snacks purchased was 0.2 (SD: 0.3) items per 100 customers in the intervention supermarkets during the intervention period. Of the intervention customers, 41% noticed the HCC and 80% of them were satisfied or very satisfied with the intervention.

**Conclusions:**

This real life experiment in supermarkets showed that the placement of healthier snacks at checkouts did not lead to the substitution of unhealthy snack purchases with healthier alternatives. Although supermarket customers positively evaluated the HCC, future studies are needed to investigate other strategies to encourage healthier food purchases in supermarkets.

## Background

Unhealthy diets are a public health concern due to the considerable contribution to the global burden of diseases [1]. Individuals with a lower socioeconomic position (SEP) especially suffer from unhealthy diets and related non-communicable diseases compared to individuals with a higher SEP [2, 3]. It is well known that the current food environment, with the extensive availability and easily accessibility of cheap, energy dense, nutrient-poor and highly processed food, is a key contributor to unhealthy food choices and consumption [4].

Supermarkets play a significant role in peoples’ food environment since they are the predominant point of purchase of foods in most affluent countries [5, 6]. In the Netherlands, more than 77% of food purchases are made in supermarkets [7]. Supermarkets use a range of in-store marketing strategies to influence food purchases of customers [8]. However, these strategies are primarily used to encourage the purchase of unhealthy, energy dense and highly processed foods [6, 9–14]. For instance, more shelf space is devoted to products such as sugary snacks and drinks than to healthy foods such as fruit and vegetables [12]. Also, unhealthy foods are often cheaper and more often heavily promoted and placed at high traffic areas such as at the end-of-aisles, shelves at eye level and at checkout counters, than healthy foods [6, 13, 14]. These insights emphasize the need for interventions that use the same strategies to stimulate healthy food choices in supermarkets [15, 16].

The checkout area is an unavoidable point of purchase in supermarkets which all customers must pass and where products are often purchased on impulse [13, 17]. Several studies demonstrated that products placed at the checkouts are predominantly unhealthy, including candy bars and sweets [13, 18–20]. In an attempt to stimulate healthy food purchases at checkout counters, the available assortment at checkouts can be optimized for health. Previous research has shown that increasing the availability of healthy foods at checkout counters in supermarkets increased the sales of these products [21–24]. However, few studies have investigated if the placement of healthier snacks at checkouts also steers customers towards the healthier over the unhealthy choice in supermarkets.

The aim of this real life experiment was to investigate if unhealthy food purchases at checkout counters in supermarkets in deprived urban areas in the Netherlands can be discouraged by the introduction of the Healthy Checkout Counter (HCC). The HCC was an initiative of a leading supermarket chain in the Netherlands and consisted of displays with a selection of healthier snacks that were placed at the checkouts, while the unhealthy snacks remained in place. In addition, we examined customers’ perceptions towards the HCC.

## Methods

### Context and the healthy checkout counter intervention

This study was conducted as part of a collaboration between the Amsterdam Healthy Weight Programme [25], the Amsterdam Health & Technology Institute (AHTI), Albert Heijn -the supermarket chain with the largest market share in the Netherlands- and the Vrije Universiteit Amsterdam in the Netherlands. The overall aim of this collaboration is to create a healthier food environment for families in deprived neighborhoods in Amsterdam and to study the effect of this effort.

In November 2015, the supermarket chain developed and implemented the HCC in 500 supermarkets spread throughout the Netherlands. Their goal was to stimulate healthy food choices at the checkout counters. For the HCC, displays were designed to be placed at the end of the conveyor belt, in front of the checkout counters, while the usual unhealthy snacks (e.g. candy bars and sweets) at the checkouts remained for sale above the conveyor belt. The displays offered a selection of healthier snacks. The healthier snacks were selected by the supermarket chain and consisted of ready to go healthier snacks with a high turnover (e.g. pieces of fruit, pre-packed vegetables, bottled water) that were already available as well as newly introduced healthier snack items specifically developed for the HCC (e.g. healthier nut bars, cereal bars, smoothies, sliced and pre-packed fruit and vegetables). In each supermarket, ~ 50% of all the checkouts contained a HCC display.

### Study design

We used a quasi-experimental design with an eight-week control period and an eight-week intervention period including fifteen intervention supermarkets in deprived urban areas in the four major cities in the Netherlands (Amsterdam, Rotterdam, Den Haag and Utrecht) that introduced the HCC and nine matched control supermarkets spread throughout the Netherlands that did not introduced the HCC. To investigate the change in purchased unhealthy snacks at checkouts between the intervention and control period, we examined the sales data of the unhealthy snacks in the intervention and control supermarkets. In addition, to explore the success of the newly introduced healthier snacks at the intervention supermarket checkouts, we also examined the sales data of those snacks in the intervention supermarkets during the intervention period. We were not able to calculate the change in these newly introduced healthier snacks since they were not yet present during the control period in both intervention and control supermarkets. The oral surveys were conducted in three intervention supermarkets in Amsterdam to investigate customers’ perceptions towards the HCC.

The Medical Ethics Committee of the Vrije Universiteit Amsterdam confirmed that this study did not apply to the Medical Research Involving Human Subjects Act (WMO), due to the nature of the measurements (anonymous sales data and anonymous questionnaires) and that therefore no approval was necessary. Verbal informed consent was obtained from all participants.

### Supermarket selection

The supermarket headquarters selected supermarkets with a HCC that were situated in the most deprived areas in the four major cities in the Netherlands. We focused on these four cities because the incidence and prevalence of childhood overweight and obesity are the highest in these cities across the Netherlands. Estimation of neighborhood deprivation was based on the values of immovable property in the area according to the annual measurements pursuant to the Valuation of Immovable Property Act (the WOZ value in Dutch), [26]. This act establishes how municipalities assess the value of homes and businesses in a neighborhood, which is strongly associated with the SEP of the neighborhood. Only supermarkets that were situated in neighborhoods with a low and very low immovable property value were included. Out of the 500 supermarkets that introduced the HCC, fifteen intervention supermarkets met our inclusion criteria and were included. The nine control supermarkets were matched to intervention supermarkets spread throughout the Netherlands based on neighborhood deprivation and store and sales profiles. With regard to customer evaluation, three intervention supermarkets in Amsterdam were selected based on pragmatic reasons.

### Study procedure and participants

In this study, the control period was from September 2015 to October 2015 (eight weeks), when the HCC was not yet introduced. The intervention period was from November 2015 to December 2015 (eight weeks). During the intervention period, the intervention supermarkets implemented the HCC, whereas the product assortment at the checkout area of the control supermarkets remained similar to that during the control period. Oral surveys were performed in three intervention supermarkets during a two-week time period in March 2016 by means of a questionnaire that was developed by the researchers. The HCC’s were still in use at that time. After customers paid their groceries, irrespective of the presence of a HCC display at the used checkout, all the customers that were 16 years or older were asked to participate in a short survey about purchase behavior in the supermarket. If customers agreed to participate, the questionnaire was read out to the customer, which took about three minutes. A total of 264 customers were asked to participate in this study**,** of which 134 (50.8%) were willing to complete the survey. Main reason for refusing to participate was ‘lack of time’.

## Measurements

### Sales data

Weekly sales data of the unhealthy and newly introduced healthier snacks during the control and the intervention period were provided by the supermarket headquarters. A few snacks that were part of the HCC were also available for sale at other parts of the supermarket. Due to the nature of the sales data we were unfortunately not able to distinguish between the location where products in the supermarket were chosen. For example, we could not determine whether a specific candy bar was picked at the candy aisle or at the checkout counter. Since we were merely interested in the effect of the HCC on snacks that were sold at checkouts, snacks that were available at other places in the supermarket were excluded from the data analyses (unhealthy snacks: *n* = 3, healthier snacks: *n* = 7). In total, the sales of 37 unhealthy and 32 healthier snacks were included for analyses in this study.

### Questionnaire

The questionnaire was based on questionnaires used in previous research that measured customers’ perceptions towards a healthy checkout in supermarkets and determinants of health behaviors [24, 27]. First, customers were asked to indicate how often they usually purchase any snacks placed at the checkout, including three response options (‘never’, ‘sometimes’ or ‘often’). Second, customers were asked to indicate if they had noticed something at the checkout area (yes or no) and if yes, what they had noticed (open question). Customers who had not noticed the HCC were informed about the HCC. All customers were asked to give the HCC a grade between 1-10, with higher scores indicating higher ratings. Third, customers were asked to indicate if they thought the ‘HCC would encourage them to make healthier food choices at checkout counters’, if ‘the HCC would encourage other customers to make healthier food choices at checkout counters’ and if ‘the HCC would decrease the likelihood that children would ask their parents for candy at checkout counters’. These answers were also rated on a 5-point Likert scale ranging from ‘strongly disagree’ to ‘strongly agree’. After that, customers were asked to provide additional suggestions for supermarkets to stimulate healthy food purchases (open question). Finally, demographic questions were asked, including sex (male, female) and age (youth (between 16 and 18 years), adults (age 18–55), older adults  (55+)).

## Statistical analysis

Descriptive statistics were used to examine the average sales data of unhealthy snacks in the control and intervention supermarkets during the control and intervention period. To make the supermarkets comparable, the sales data is presented as average weekly sales (items) per 100 supermarket customers. Linear regression analysis was conducted to investigate the difference in the change of sales of the unhealthy snacks at checkouts between the control and intervention supermarkets in the control and intervention period. Condition (intervention =1, control = 0) was used as independent variable and sales of unhealthy items per 100 customers per week as the dependent variable. Furthermore, descriptive statistics were used to examine the sales data of the healthier snacks during the intervention period in the intervention supermarkets. In addition, we assessed the five healthier snacks most frequently sold at checkouts during the intervention period in the intervention supermarkets. The 5-point Likert scale response categories of the questionnaire data were recoded from to − 2 to + 2. Descriptive statistics were used to summarize the demographic variables of the study sample and to gain insight in customers’ HCC evaluation for customers that noticed the HCC and those that did not separately. We used a nominal significance level of 5% to assess statistical significance and all analyses were performed by using R version 3.5.1.

## Results

### Sales data

**Fig. 1:**
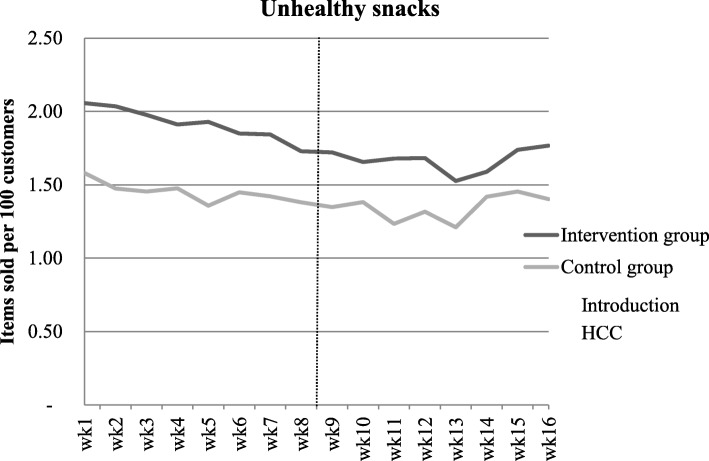
Weekly sale figures of the unhealthy snacks for the control and intervention supermarkets during the control and intervention period

As presented in Fig. 1 the average sales of unhealthy snacks in the intervention supermarket was 1.9 (SD: 0.1) items per 100 customers during the control period and 1.7 (SD: 0.1) items during the intervention period (change: − 0.2 items per 100 customers). The average sales of unhealthy snacks in the control supermarket was 1.5 (SD: 0.1) items per 100 customers during the control period and 1.4 (SD: 0.1) items during the intervention period (change: − 0.1 items per 100 customers). The linear regression analysis showed no statistically significant difference in the change of sales of the unhealthy snacks at checkouts between the control and intervention supermarkets (B = − 0.008. 95% CI = − 0.154 to 0.139, Table 1).

**Table 1:** Change in sales of the unhealthy snacks at checkouts between the control and intervention supermarkets in the intervention and control period

Supermarket	Beta (95% CI)	Beta (95% CI)	t-value
Intervention supermarkets Control supermarkets	−0.08 (− 0.13, − 0.02) −0.07 (− 0.14, 0.00)		−2.88 −2.18
Intervention vs. control supermarkets Intercept		−0.01 (− 0.15, 0.14) 0.06	−0.12

*CI* Confidence interval

**Fig. 2:**
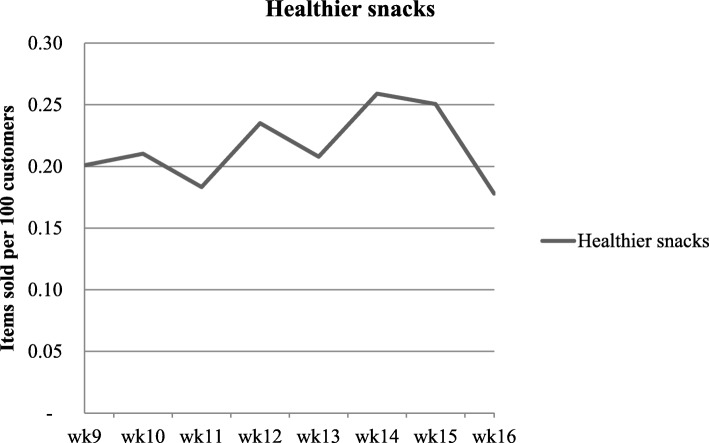
Weekly sale figures of the healthier snacks for the intervention supermarkets during the intervention period

The sales of the newly introduced HCC healthier snacks was on average 0.2 (SD: 0.3) items per 100 customers (Fig. 2). The five HCC healthier snacks that were sold most often were pre-packed snack tomatoes (14.6%) and four different nut and cereal bars (28.9%).

### Oral surveys

Of the 134 customers, 58% were women and most were adults (18-55y: 65.2%) or older adults (>55y: 23.9%). Most of the customers indicated to ‘never’ purchase foods at checkouts (61.2%) (Table 2). Almost two third of the customers did not notice the HCC (65.7%). We observed small differences in satisfaction and appreciation of the HCC between customers who noticed the HCC and those who did not. Of those who noticed the HCC, 80% was satisfied or very satisfied with the intervention, vs. 77% for those who did not notice the HCC, but to who the HCC was explained. The average grade of the HCC was 7.4 out of 10 (SD = 1.2) for those who noticed the HCC and 7.2 out of 10 (SD = 1.3) for those who did not notice the HCC (Table 3). The three most frequent suggested ideas for supermarkets to stimulate healthy food choices from the overall sample were that supermarkets 1) should increase the promotion of healthy foods (29.9%), 2) reduce the prices of healthy foods (26.1%) and 3) remove unhealthy snacks at checkout counters (18.7%).

**Table 2:** Characteristics of customers of the three participating supermarkets who completed the survey (*n* = 134)

	n (%)
Total	134 (100)
Sex	
Women	78 (58.2)
Age (category)	
Youth (16 < 18 year)	16 (11.9)
Adults (18–55 year)	86 (64.2)
Older adults (> 55 year)	32 (23.9)
Habitual purchase frequency at the checkout	
Never	82 (61.2)
Sometimes	30 (22.4)
Often	22 (16.4)
Number of participating customers per supermarket	
Supermarket 1	49 (36.6)
Supermarket 2	44 (32.8)
Supermarket 3	41 (30.6)

*N* Number

% percentage

**Table 3:** Perceptions towards the Healthy Checkout Counter (HCC) of customers of the three participating supermarkets apart from customers that noticed the HCC and customers that did not notice the HCC (*n* = 134)

		Customers that did not notice the HCC (*n* = 79)	Customers that noticed the HCC (*n* = 55)
Grade	M (SD)	7.2 (1.31)	7.4 (1.20)
Satisfaction with the HCC	*n* (%)		
very unsatisfied		2 (2.5)	1 (1.8)
not satisfied		1 (1.3)	2 (3.6)
neither unsatisfied nor satisfied		15 (19.0)	8 (14.5)
satisfied		39 (49.4)	25 (45.5)
very satisfied		22 (27.8)	19 (34.5)
Expected positive effect of the HCC on own purchased foods at the checkout counter	*n* (%)		
strongly disagree		16 (20.3)	11 (20.0)
disagree		24 (30.4)	11 (20.0)
neither disagree nor agree		12 (15.2)	11 (20.0)
agree		26 (32.9)	18 (32.7)
strongly agree		1 (1.3)	4 (7.3)
Expected positive effect of the HCC on purchased foods of other customers at the checkout counter	*n* (%)		
strongly disagree		2 (2.5)	1 (1.8)
disagree		5 (6.3)	1 (1.8)
neither disagree nor agree		19 (24.1)	14 (25.5)
agree		50 (63.3)	28 (50.9)
strongly agree		3 (3.8)	11 (20.0)
Expected positive effect of the HCC on children asking for candy at the checkout counter	*n* (%)		
strongly disagree		14 (17.7)	11 (20.0)
disagree		22 (27.8)	9 (16.4)
neither disagree nor agree		9 (11.4)	13 (23.6)
agree		29 (36.7)	14 (25.5)
strongly agree		5 (6.3)	8 (14.5)

*M* Mean

*SD* Standard deviation

N Number

% Percentage

## Discussion

This study showed that the placement of healthier snacks at checkouts did not lead to the substitution of unhealthy snack purchases with healthier alternatives. The customer evaluation showed that although a majority of customers did not notice the HCC, they positively evaluated the checkout intervention of the supermarket chain.

With respect to the sales data, our finding that the placement of healthier snacks at checkouts did not lead to the substitution of the purchase of unhealthy snacks with that of healthier snacks, is in line with previous studies on healthier product placement at checkout counters in supermarkets [21, 22]. For instance, a previous study in the United States showed that the additional placement of lower calorie beverages and water in checkout coolers with sugary drinks in supermarkets, increased the purchase of water, whereas the sales of the sugary drinks remained stable [21]. Two other previous studies found that the removal of unhealthy snacks from a checkout and their replacement with healthier snacks increased the purchase of these healthier snacks in supermarkets [23, 24]. However, these studies included merely one checkout per supermarket and no intervention effect on the sales of the unhealthy snacks at the other checkouts was found. The results of our study and these earlier studies together, suggest that in order to promote healthy food choices and to discourage the impulsive buying of unhealthy snacks, supermarkets should substitute the total unhealthy snack assortment with healthy alternatives at all the checkouts.

The insignificant effect of the HCC on the purchase of unhealthy snacks may be explained by a number of factors. First, merely ~ 50% of the checkout counters in the intervention supermarkets were provided with the HCC displays with healthier snacks. As a result, a group of customers were unexposed to the HCC during the intervention period and therefore the exposure could be too limited to cause an effect detectable in the sales data. This was assumed by another study that did not find an effect of a healthy checkout counter on the sales of unhealthy snacks [24]. In this previous study, healthy snacks were provided at only one supermarket checkout counter so customers had limited exposure to healthy snacks. Second, in the HCC a smaller number of healthier snacks was available compared, to the large variety of unhealthy snacks that was available from the displays above the conveyor belt at checkouts. It could be that the exposure of healthier snacks vs. unhealthy snacks was too low to cause and effect in the purchase of unhealthy snacks. Moreover, the HCC displays were placed in front of the checkouts, while the unhealthy snacks were placed above the conveyor belt. Consequently, customers were exposed to the unhealthy snacks longer than they were to the healthier snacks from the HCC. It could also be that the results of our study are due to a floor effect, given that the sales numbers of unhealthy products at the checkout per customer were quite low to begin with, and that we may have found a larger effect in supermarkets with higher sales of products at the checkout. However, our study is limited to supermarkets in disadvantaged areas and it could be that the sales at checkouts in supermarkets in less deprived areas is higher. Although we can only speculate about the effects of the HCC in supermarkets in areas with a higher SEP it could be that the sales of checkout counter snacks in these supermarkets are higher compared to those in areas with a low SEP since single item snacks at check outs are most often more expensive than multi-pack snack items. If there is a potential floor effect in our study than the results could be different in supermarkets in areas with a higher SEP. On the other hand, customers with lower SEP may be relatively less health conscious compared to those with higher SEP and the purchase of unhealthy products at the counter may be relatively high.

With regard to the customer evaluation, we observed no differences in the evaluation of the HCC between customers that noticed the HCC and those that did not, but were informed about the initiative during the survey. In line with prior studies [18, 24, 28, 29], our results showed that, regardless whether changes within supermarkets are observed or not, customers are supportive of in-store interventions that encourage healthy food choices and will be accepted by customers. This was also confirmed in a previous Dutch study on nudging healthy food choices at checkouts in kiosks at a train station where the majority of customers was positive about the initiative and accepted the nudge, whereas 75% of those customers did not notice the nudge [30]. In addition, another Dutch study showed that disclosing the intended purpose of a nudge to promote healthy food choices did not interfere with its effects; the increased sales of healthy snacks as a result of the nudge remain robust when customers are made aware of the nudge [31]. This may be relevant to the debate regarding the ethics of implementing nudging interventions that influence individuals without their awareness. Our and these previous findings together suggest that it is possible for supermarkets to nudge customers, without customers feeling manipulated or having their freedom of choice threatened while being nudged.

Our findings should be considered in the light of the following strengths and limitations of our study. Strengths include the relatively large number of supermarkets included and the use of both objective measures of food purchases as well as subjective customer perceptions surveys to evaluate (the effectiveness of) the HCC. Moreover, we studied the effects of the HCC in a real life setting and the HCC was developed and implemented by the supermarket chain and therefore perceived as a feasible intervention to be implemented and scaled by the supermarket itself. However, this resulted in the limitation that we could not control the intervention development and its implementation, which had an impact on the choice of the healthier snacks offered by the HCC. The healthier snacks that were developed by the supermarket chain were lower in sugar and saturated fat than the unhealthy snacks at the checkout. However, a part of those snacks still did not meet the dietary recommendations the Dutch Nutrition Centre [32]. Finally, we did not assess the fidelity of the execution of the intervention to examine to which extent the intervention was implemented as intended by supermarkets. Therefore under- and overestimation of the effect of the HCC cannot be excluded.

Since our study showed that the placement of healthier snacks at checkouts did not lead to the substitution of unhealthy snack purchases with healthier alternatives, future studies should investigate the short- and long-term effects of substituting the total unhealthy snack assortment at checkout counters with exclusively healthy snacks. Furthermore, it is questionable if a healthy checkout area alone can improve overall healthy food purchases in supermarkets, since 70% of the food products that are sold in supermarkets are unhealthy, highly processed foods [33, 34]. To achieve a larger impact on overall healthy food purchases, future studies should incorporate a healthy checkout strategy as part of a set of multiple approaches to stimulate healthy food choices in supermarkets. This was also confirmed in previous research that suggested that the most effective strategies should be combined in health interventions and implemented simultaneously to stimulate overall healthier food purchases in supermarkets [16, 35, 36].

## Conclusion

Our study showed that the placement of healthier snacks at checkouts did not lead to the substitution of unhealthy snack purchases with healthier alternatives. These results suggest that the HCC is not effective in decreasing unhealthy food purchases at supermarket checkouts. Future research should study the short- and long-term effectiveness of substituting unhealthy snacks with exclusively healthy snacks at the checkout counter and should combine this with other in-store interventions.

## Data Availability

The datasets generated and/or analyzed during the current study are available from the corresponding author on reasonable request.
